# Assessing the ecosystem services provided by urban green spaces along urban center-edge gradients

**DOI:** 10.1038/s41598-017-11559-5

**Published:** 2017-09-11

**Authors:** Jie Chang, Zelong Qu, Ronghua Xu, Kaixuan Pan, Bin Xu, Yong Min, Yuan Ren, Guofu Yang, Ying Ge

**Affiliations:** 1 College of Life Sciences, Zhejiang University, 866 Yuhangtang Road, Hangzhou, 310058 China; 2 School of Economics, Zhejiang Gongshang University, 18 Xuezheng Street, Hangzhou, 310018 China; 3 College of Computer Science, Zhejiang University of Technology, Hangzhou, 310024 China

## Abstract

Urban green spaces provide various ecosystem services, especially cultural services. Previous assessment methods depend either on hypothetic payments for ecosystems or real payments not directly related to ecosystems. In this paper, we established a method for assessing the cultural ecosystem services in any location in urban area using only two variables, green space (ecosystem) and land rent (real payment). We integrated the cultural and the regulating services into the total ecosystem services because urban green spaces provide almost no provisioning services. Results showed that the same area of green spaces near the center provided much higher cultural services than that near the urban edge; the regulating services accounted for 5% to 40% of the total ecosystem services from the center to the edge of urban area; along the center-edge gradient, there was a threshold out which the ecosystem services were lower than the maintenance cost of green spaces.

## Introduction

**Figure 1:**
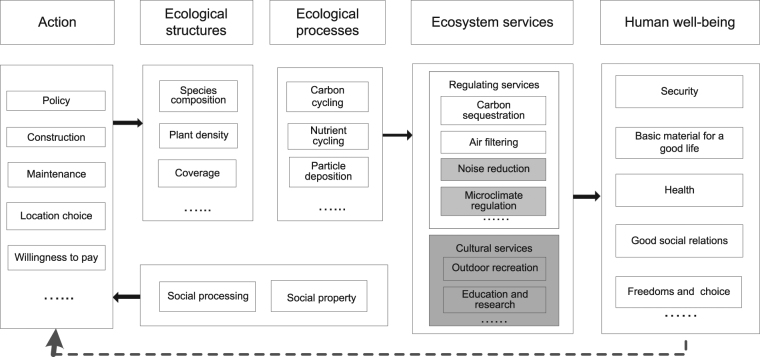
Framework for action-feedback loop between human actions and well-being. This framework integrated elements in some existing frameworks^9, 43, 44^. Gray-filled boxes represent the services that can be perceived directly by humans, white boxes represent services that cannot be perceived directly by people. Solid arrows represent influences, and the dotted arrow represents feedback from human well-being to action by people concerning green spaces. Detailed items of ecossystem services are showed in SI.

Urban green spaces are expanding faster than the population that is quickly increasing in urban areas in recent decades^1, 2^. The requirement of ecosystem services provided by urban green spaces is increasing greatly^3, 4^, but assessing ecosystem services is still a challenge. Urban green spaces provide greater amounts of cultural services^5, 6^, for example spiritual and religious, recreation and ecotourism and aesthetic^7, 8^, than other ecosystem services. In contrast, forests in rural areas provide a considerable amount of provisioning services, for example wood and fiber^9^ that almost are not provide by urban green spaces^10^. Urban green spaces provide considerable regulating services^11^, but most of them cannot be perceived directly by people (Fig. 1). Then three essential questions arose^12^, including what kind of ecosystem services could be accomplished? Where should green spaces be located? How could high ecosystem services be achieved?

Cultural services of urban green spaces can be perceived and are the main contributors to human well-being for urban inhabitants^7, 13, 14^. For cultural services to be beneficial, individuals must be in the vicinity^15, 16^ such that the spatial distribution of ecosystem services provided by green spaces are crucial for the unevenly distributed population in the urban area^17^. An average value of cultural services can hardly explain why green spaces are constructed in prized urban areas, especially near the city center, where land prices are so high. Unfortunately, a method for proper assessment of the spatial pattern of ecosystem services provided by urban green spaces is lacking. Without sufficient information, urban managers cannot carry out a cost-benefit analysis for green spaces planning and management.

Previous methods assessing cultural services depend on either hypothetic payment for ecosystems or real payment but not for ecosystems. The major one is the hedonic pricing method (HPM) based on real payment of the selling price of houses near the green spaces^18, 19^. Another is the contingent valuation method (CVM) that assesses the cultural services of green spaces based on the willingness of people to pay rather than a real payment behavior^9, 20^. However, the cultural services derived by the HPM are not ecosystem-based, HPM is housing-based and measure the extra profits related to the green spaces nearby (Supplementary Fig. S1). Therefore, the cultural services by HPM cannot be integrated with the regulating services into the total ecosystem services for the basics are different. In addition, HPM requires many variables such as building ages and surrounding environment factors and complex calculations.

**Figure 2:**
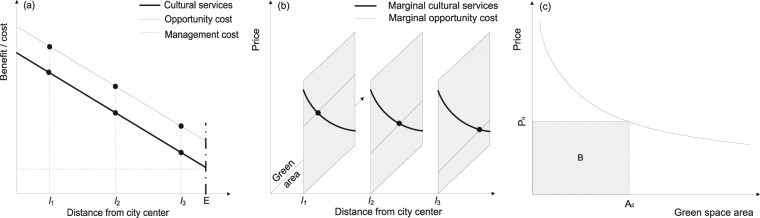
Benefit and cost of green spaces along a center-edge gradient in the urban area. (**a**) Spatial pattern of perceived benefits (cultural services), land rents and management costs along the gradient, for example locations *l*_1_, *l*_2_ and *l*_3_. Letter E on x-axis represents the edge of urban area in a city. (**b**) Marginal benefits (bold lines) and marginal costs (thin lines) along the center-edge gradient and there is an equilibrium point in any location *l*. (**c**) A heuristic model of perceived ecosystem services (mainly cultural services) in a location *l*, and P_0_ represents the satisfaction of human preferences derived from given area of green spaces (A_0_) in monetary units, the grey shadow B denotes the benefits of people from the perceived ecosystem services.

In this study, we developed a method that we called the land rent method (LRM) to measure the real payment based on the ecosystems to analyze the ecosystem services across the entire urban area (Fig. 2). The LRM employs two effective variables, the land rent and the coverage of green spaces in any locations in urban area. We chose three case cities (Supplementary Fig. S2), Beijing, Guangzhou and Hangzhou, to carry out the investigations for spatial patterns of land rent and green spaces. Finally, we obtained the cultural services based on both land rent and green space coverage in each location from the center to the edge of urban built-up areas. The regulating services and cultural services in each location were further integrated into the total ecosystem services, which were used for the cost-benefit analysis of green spaces in any location across the urban area.

## Development of the Land Rent Method

In urban areas, people perceive the cultural services^6^ and a few regulating services (such as microclimate regulation and noise mitigation) directly, and the cultural services should be the major feedback variable for whether the public supports urban green spaces or not^8^. Therefore, assessing the cultural services in an urban area can help realize the ‘action-feedback chain’ in a self-organized city (Fig. 1). The action-oriented framework^21^ that combines cultural services and regulating services as the total ecosystem services of green spaces can explain the self-organization mechanism of a city.

Developing an effective assessment method for cultural ecosystem services provided by urban green spaces requires finding out the key variables that are critical to an urban ecosystem. Basically, land rent is the opportunity cost for a land use, for example, urban green spaces, roads and a shop. Land rents reflect the lost benefits (e.g. business building, residential building) that the public has to bear for an alternative land use (green spaces). Green space coverage is a classical variable in ecology^1^ and the most visualized variable for cultural services^20, 22^, and is also a key variable for regulating services^11^. The spatial pattern of green spaces indicates the diverse preferences of people for cultural services in different locations in urban areas (Fig. 2a).

**Figure 3:**
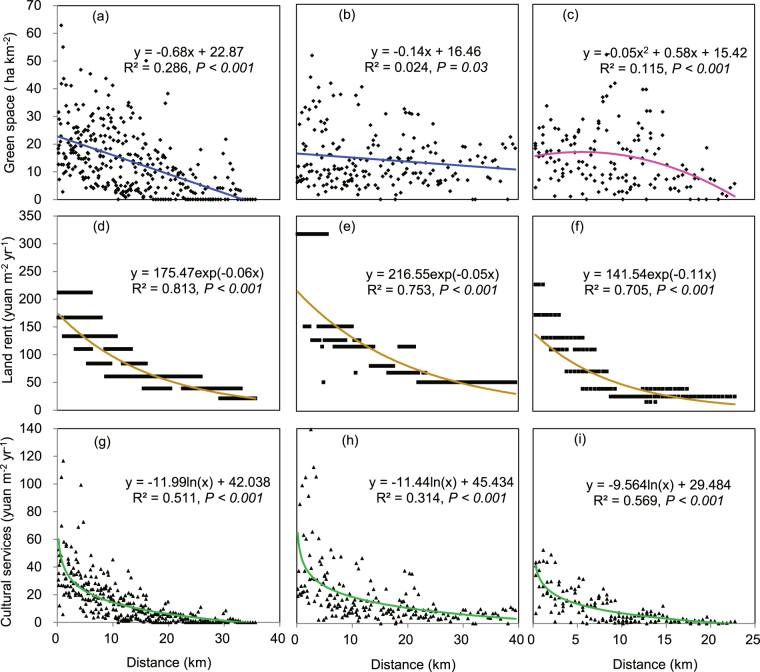
Spatial patterns of green space coverage, land rent and cultural services in the center-edge gradients of urban areas. (**a**–**c**) Green space coverage in Beijing, Guangzhou, and Hangzhou, respectively. (**d**–**f**) Land rents in Beijing, Guangzhou, and Hangzhou, respectively. (**g**–**i**) Cultural services in Beijing, Guangzhou, and Hangzhou, respectively. Green lines indicate negative logarithmic patterns, pink line indicates quadratic pattern, and brown lines indicate negative exponential patterns, blue lines indicate linear patterns.

Here, we focus only on the green spaces that are open to the public. We assume that: (1) the public constructs and maintains green spaces based on the value of perceived ecosystem services, which are mainly cultural services (Fig. 2b); (2) the green space coverage in a location is the trade-off of benefits (perceived ecosystem services) and costs (Fig. 2c); (3) the land rents are the opportunity cost paid by people, who are the land owners as well; and (4) the green spaces as a public good is not profitable, in other words, the benefits are equal to the cost.

Green space coverage (*CG*_*l*_) in a location *l* was calculated as,

1 $$C{G}_{l}=A{G}_{l}\times {M}_{l}/{A}_{l}$$

where *AG*_*l*_ is the projected area of green spaces in *l*, *A*_*l*_ is the area of each quadrat in *l*, *M*_*l*_ is a modifying factor reflects the height and density of green spaces (here we set it as 1 to simplify calculation).

The land rent in a location *l* (*LR*_*l*_) is calculated by the land price (*LP*_*l*_) and time duration (*t*) of land use right transfer,

2 $$L{R}_{l}=L{P}_{l}/t$$

Due to green spaces being non-profit public goods, the marginal benefit in a location *l* (*MB*_*l*_) equals to the marginal cost (*MC*_*l*_). *MB*_*l*_ is the increment of perceived services along with the increasing green space coverage in a location *l*, and *MC*_*l*_ is the opportunity cost in *l* indicated as land rent.

The cultural services provided by green spaces in *l* (*CES*_*l*_) can be calculated as,

3 $$CE{S}_{l}=(L{R}_{l}\times A{G}_{l}\times {M}_{l})/{A}_{l}-PRE{S}_{l}=L{R}_{l}\times C{G}_{l}-PRE{S}_{l}$$

where *PRES*_*l*_ is the perceived regulating services, which are noise reduction and microclimate regulation in this paper. The perceived regulating services have been eliminated to avoid double counting.

The eq. (3) describes the land rent method (LRM), which utilizes the actual payment of public, for assessing the cultural services provided by green spaces. The method can be applied at a fine scale over an entire urban area.

## Results

According to the LRM, the cultural services of green spaces in a specific location are determined by the coverages of green spaces and the land rent in the location. The field investigation showed that the spatial patterns of the coverage of green spaces were different in the three cities. In Beijing city and Guangzhou city, the coverages of green spaces decreased linearly along the gradient from city center to urban edge (Fig. 3a and b), while in Hangzhou city (Fig. 3c), the coverages followed a quadratic trend along the gradient. Different from the patterns of green spaces, the land rents in the three case cities showed monotonic decreasing trends from the city center to the edge of the urban area, and they followed the exponential functions in all three cities (Fig. 3d–f).

**Figure 4:**
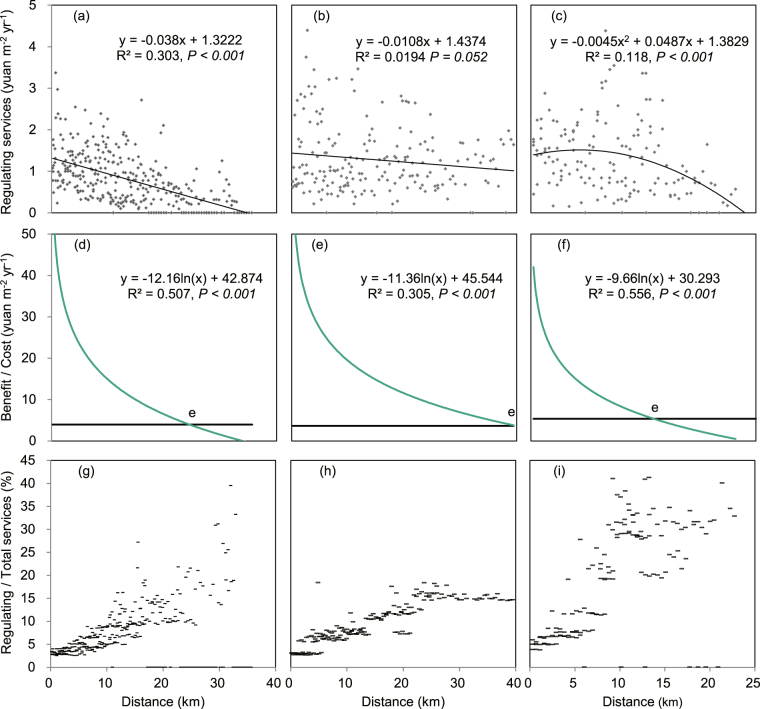
Cost-benefit analysis of green spaces along the center-edge gradients of urban areas. (**a**–**c**) The regulating services in Beijing, Guangzhou, and Hangzhou, respectively. (**d**–**f**) The total ecosystem services (green lines) and the management costs (dark lines) in Beijing, Guangzhou, and Hangzhou, respectively. (**g**–**i**) The proportion of regulating services in total ecosystem services in Beijing, Guangzhou, and Hangzhou, respectively. The letter (**e**) represents the intersection of total services and management costs.

The cultural services provided by green spaces in a specific location were showed as a point in Fig. 3(g–i). The regression analysis showed that the cultural services provided by the green spaces are monotonic decreasing from the city center to the edge of urban area and followed a logarithmic function in all three case cities (Fig. 3g–i). The regulating services of green spaces were only determined by the green spaces in a specific location. The calculated results performed as the points in Fig. 4a–c. In Beijing city, the regulating services were declining while in Hangzhou they climbed up and then declined from the center to the edge of the urban areas (Fig. 4a and c), but there was no trend in Guangzhou (Fig. 4b).

As mentioned before, the total ecosystem services provided by urban green spaces include only the cultural and regulating services. It is noted that the regulating services include both the perceived regulating services and other non-perceived services (for example carbon sequestration, oxygen release and biogenic volatile organic compound emissions, details in Supplementary Table S1). The total services in each location of the three cities were showed as the points in Fig. 4d–f. The statistical spatial patterns of the total ecosystem services in all the three case cities followed negative logarithmic functions (Fig. 4d–f).

The proportions of the regulating services in the total services along the center-edge gradients increased from lower than 5% near the center to more than 40% near the urban edge (Fig. 4d–f). The average proportions of regulating service in the total ecosystem service of the green spaces were 8%, 9%, and 17% in Beijing, Guangzhou and Hangzhou city, respectively; the cultural services contributed the most to total ecosystem services in urban areas.

The green spaces’ management costs, which include construction and maintenance costs of green space, were assumed constant along the center-edge gradient. An intersection point (e in Fig. 4d–f) of management costs and total ecosystem services occurred at a particular location in each of the three cities. Between the intersection point and the city center, the benefits (total ecosystem services) of green spaces are higher than the management costs. The intersection point in Beijing was 25 km from the center (Fig. 4a), in Guangzhou it was 40 km from the center, and in Hangzhou it was only 15 km (Fig. 4c). Outside the intersection points in these cities, the total ecosystem services provided by the green spaces were lower than the maintenance costs. In other words, the benefit is lower than the management cost near the urban edge.

The spatial patterns of cultural services among the three case cities performed scale dependence: the intercepts of the functions showed that the cultural services near the center of a bigger city (Beijing or Guangzhou) are higher than that of a smaller city (Hangzhou); the coefficients of the functions performed that the rate of outward attenuation in a bigger city is slower than that in a smaller city.

## Discussion

**Figure 5:**
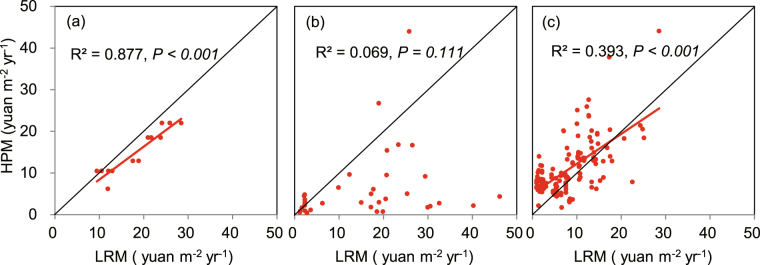
Comparison of the cultural services assessed by land rent method (LRM) and holenic pricing method (HPM)^45^. Red dots represent the matched-pair comparison between values gotten from LRM and HPM at the same location in (**a**) Beijing, (**b**) Guangzhou and (**c**) Hangzhou. Red lines indicate the linear regression, black lines show the 1:1 line.

To evaluate the reliability of this new method, we compared the results obtained by the LRM with those obtained by the HPM at the same locations along the urban-edge gradients in a city. General regression models were employed to realize the comparison. In Beijing, the results exhibited LRM vs. HPM slopes from 0.56 to 0.99 with an average value of 0.79 (Fig. 5a). In Guangzhou, a nonsignificant relationship existed (Fig. 5b). The LRM offered relative higher values than that of HPM in most points. In Hangzhou, there was a similar range of slopes from 0.56 to 0.87 with an average value of 0.72 (Fig. 5c). In Beijing and Hangzhou city, the LRM and the HPM showed a remarkable corresponding relationship (Fig. 5). It suggests that the LMR is useful for the assessment of the cultural services provided by green spaces. We did not compare the results by LRM with those by CVM due to the very small datasets of CVM in the three cities.

The advantages of using the LRM to assess the spatial pattern of cultural services in urban areas include at least three aspects. First, LRM has a solid dataset foundation for green space coverage and land rents are available across urban areas. The land price or rent is a classical variable in economics and is obtainable in many urban locations from census data worldwide^23, 24^. The large datasets make the results obtained by LRM more objective than other methods that are based on smaller datasets (Supplementary Fig. S1). Second, the LRM measures the ecosystems (green spaces) directly; the cultural services and regulating services are comparable and can be integrated to the total ecosystem services in any location. With awareness of the ecosystem services in a location, people can understand the benefits (ecosystem services) they obtain from the green spaces, and give feedback of their willingness to urban planning for tradeoffs between green spaces and other land uses in a location of the urban area. Third, using fewer variables makes the LRM easy to use and having lower system error than other methods. There may be concern about an endogeneity problem between land rent and green spaces, but land rent and green spaces are independent variables, for land rents in a location are determined mainly by the distance from the city center while natural amenities have no effect on urban land prices at a large scale^25^. In sum, the LRM has potential to become a general assessment method of ecosystem services.

The decreasing patterns of cultural services of green spaces (Fig. 3g–i) match the demand patterns for cultural services per land area being higher near the center, where there is higher population density^26^, than near the edge of the urban area^4, 27, 28^. It has been demonstrated that population density has a positive relationship with green space value^29^. In addition, the perception of cultural services per land area near the center is also higher than that near the urban edge because the use frequency of green spaces is correlated positively with accessibility^30, 31^.

The spatial patterns of green space coverage were diverse in the different cities. In Beijing and Guangzhou, the decreasing trends existed, similar to those in some cities such as Leipzig^32^, while in Hangzhou (Fig. 3a–c) a quadratic pattern existed, similar to those in other cities such as Minneapolis^33^. When the spatial patterns of land rent were compared to the green space coverage, we concluded that the cultural services were determined mainly by the land rent.

As a determined variable of the cultural services, the land rents (or land prices) are negatively exponentially decreasing from the center to the edge of the urban area (Fig. 3d–f) in this study and in 35 other cities^34^. In addition, we found that the spatial pattern of land rent in a city were size dependent (Fig. 3), the exponential pattern of land rent flattened (Supplementary Fig. S3A and B) in cities with bigger population in comparison to cities with smaller population. It is predicted that the cultural services of green spaces would also flatten out with cities getting bigger.

The spatial pattern of land rent in urban area is also changing with the development of a city. When re-compiling the data from literature^35^, we found that the exponents of the functions for land rent patterns during 1835 to 1990 in New York had been changed from −0.39 to −0.06 (Supplementary Fig. S3C and D). It means that the land rent is flattening along with the development of New York. It is noted that the exponentials in Beijing and Guangzhou were −0.06 and −0.05, respectively, which were quite close to that of New York in 1990. In light of the LRM, it is predicted that the spatial patterns of the cultural services of green spaces may become more homogeneous along with urban development. The flattening land rent pattern indicated that evenly distributed green spaces generate cultural services for urban inhabitants along with the progress of social-ecological systems.

The spatial patterns of regulating services in the case cities were similar to the patterns of green space coverage since regulating services rely mainly on the biomass and productivity of vegetation^11^. The low percentages of regulating services in the total ecosystem services (Fig. 4g–i) verifies the hypothesis that cultural services are the main ecosystem service provided by green spaces in the urban areas^7, 8^. We further identified that cultural services contribute the most of the ecosystem services near the city center, but the cultural services and regulating services were similar closer to the edge of urban areas.

The results of cost-benefit analysis based on the ecosystem services and management suggest that the quality of green spaces should be considered in policy making. For example, improving the quality (better species and optimized community structure) near the city center, while chosing some low-cost species and apply simple management near the urban edge would made more economic sense.

Until now, we have answered the above mentioned three essential questions^12^ of ecosystem services accomplished from urban green spaces. We demonstrated that the cultural services are the major feedback variables of the public supporting green spaces distributing in urban areas (Fig. 1). We also found that a unit green space near the city center provides much higher cultural services than those near urban edge (Fig. 3g–i) therefore more green spaces should be built near the center than near the urban edge to achieve higher cultural services. In addition, the monotonic decrease of the benefit (Fig. 4d–f) along the center-edge gradient suggests that policy makers have to consider the differences of the scarcity of the cultural services between the center and the edge of urban areas in urban planning and management. For example, near the city center, the benefits of green spaces were more than 10 times than their cost in Beijing and Guangzhou and more than 5 times in Hangzhou; while near the edge (10 km away from the city center), the benefits were 4, 5, and 2 times in Beijing, Guangzhou, Hangzhou, respectively. It implied that green spaces were scarcer near the city center than that near the edge. Nevertheless, in Hangzhou, the famous scene West Lake, which occupies 6.5 km^2^ areas near the city center, satisfied a part of cultural service need and thus reduced the scarcity of green spaces.

There are several limitations of the LRM. First, in some cities, the remnant vegetation (similar to rural vegetation) provides high cultural services for a specific purpose such as the relational values of old trees^36^, but LRM cannot assess the variance currently for we assume the influence factor as 1 for simplicity. Second, the LRM uses the benchmark land price to calculate the land rent, but the grain of benchmark land rents are coarse than the sample of green spaces. It may weaken the credibility of the nonlinear pattern of cultural services (Fig. 3g–i). When applied spatial autocorrelation (Moran’s *I*) in ArcGIS^37^ to analyze the interferences of different variances between green spaces and land rents, we found that both the green spaces and land rents are clustered in Beijing and Hangzhou, while those are random in Guangzhou. The consistency of spatial agglomeration excluded the effects of the differences between the grain of land rent and green space on the nonlinear pattern. Although the transaction land price is often used in the land market^24^, the transaction land price is temporary and volatile^38^ and a complete dataset for a whole city is almost inaccessible. However, the benchmark land price (or land base value) datasets are available in many cities worldwide^23^, meaning that land rent based on benchmark land price is feasible and representative.

## Conclusions

To our knowledge, this is the first study on the cultural services of green spaces along the center-edge gradient of an urban area and the spatial relationship between cultural services and regulating services. The LRM is a new and effective tool for assessing cultural services of urban green spaces. With the LRM, we can identify different economic values with similar biophysical values (such as biomass and coverage) in different locations, and carry out the cost-benefit analysis of maintaining or expanding green spaces along the center-edge gradient for the sustainable development of cities. Our findings suggest that more green spaces should be built near the center for achieving higher cultural services in than near the edge with lower land rent in urban area.

The new method contributes to the development of urban ecology. The LRM integrates both ecological and economic factors, and helps the methodology in urban ecology shifting from ‘ecology in the city’ to ‘ecology of the city’. We see our method as an important step towards a unified urban ecology theory to find approaches for improving human well-being in a quickly urbanizing era.

## Methods

We applied the LRM in three Chinese cities: Beijing, Guangzhou, and Hangzhou. We chose these cities for three reasons: first, the coverage of green spaces in each city is moderate (the coverages are 42%, 36%, and 37% in 2005, respectively); and second, the three case cities have mature land markets, from which we can obtain representative land price gradients; third, the population of these urban areas is of different size ranging from 4 million to 11 million.

### Investigations for green spaces

Through visual interpretation based on remote-sensing imagery from Google Earth, we established four transects in Guangzhou and Hangzhou, and six transects in Beijing because of the huge built-up area and population, radiating from the city center to edge (Supplementary Fig. S2). In each transect, we delineated quadrats of 450 × 450 m as the study quadrat^38, 39^. We excluded the water bodies and remnant forests in this study. We identified the green spaces using the polygon tool in Google Earth and calculated the area of the green spaces inside the quadrat. We drew the distributions of green spaces after measuring their area in each quadrat along the gradient combining with the location of each quadrat.

We conducted a field survey of the green spaces to obtain the parameters, including plant species, plant density, diameter at breast height (DBH, 1.3 m from the ground) of trees, and so on. We conducted the 400 m^2^ quadrats to obtain the parameters of the community structure in green spaces following common used method^40^, and used a stratified random selection method within the built-up area^41^ in the sample transects. The center point of each quadrat was determined by Google Earth (version 6.2.2.6613), and a GPS was equipped for field positioning. In each quadrat, we identified tree species and measured tree height, DBH, tree ring at DBH, crown shape, crown width, canopy cover area, and the coverage of shrubs and herbs.

### Investigations for land rents

We obtained the commercial benchmark land price map (for example, Supplementary Table S2) from the Land Resources Bureau of the city or China’s urban land price monitoring network^42^. We used the quadrats set for identifying green spaces from the city center to edge. According to the land price map or document, we obtained the land price (yuan m^−2^) of each sample point (1 yuan = 8.28 USD in 2004). The projected coordinates and the land price information of sample points were analyzed in ArcGIS 10.2^37^ and exported into the Excel for processing.

### Assessment of the regulating services

We assessed the value of seven regulating services (carbon sequestration, oxygen release, air filtering, runoff mitigation, noise reduction, microclimate regulation, and biogenic volatile organic compound emissions) provided by green spaces (see more details in SI). The noise reduction and microclimate regulation were the perceived regulating services. To avoid double-counting, the values of these two regulating services were eliminated in cultural services calculation (eq. 3).

## Electronic supplementary material

Supplementary Information 
