# Supplementary material for: Assessing the ecosystem services provided by urban green spaces along urban center-edge gradients
Source: Sci Rep. 2017 Sep 11;7:11226. doi: 10.1038/s41598-017-11559-5 (PMC5593898; doi:10.1038/s41598-017-11559-5)
Supplement: Supplementary file 1 — Supplementary Information [file 41598_2017_11559_MOESM1_ESM.pdf]

Assessing the ecosystem services provided by urban green spaces along urban center-edge gradients

Supplementary information

Jie Chang[1], Zelong Qu[1], Ronghua Xu[1], Kaixuan Pan[1], Bin Xu[2], Yong Min[3], Yuan Ren[1], Guofu Yang[1], Ying Ge[1]*

*To whom correspondence should be addressed. E-mail: geying@zju.edu.cn

This file contains:

Supplementary Methods

Calculation for cultural services

Calculation for the monetary value of regulating services

Data collection and re-calculation for cultural services by CVM and HPM

Figure S1-S3

Table S1-S7

References

Supplementary Methods

Calculation for cultural ecosystem services

The green space coverage in each quadrat was calculated using Eq. (1) in the main text. We then obtained a one-dimensional scatter plot with distance from the city center as the X axis in each city. For determining the spatial patterns of green spaces along center-edge gradients, we tried several types of functions, including linear, exponential, logarithmic, and quadratic functions separately, by simple regression analyses. We chose the suitable equation according to the maximum explanatory power (coefficient of determination, r^2).

The land rent in a location (yuan m^{-2} urban area yr^{-1}) was calculated using Eq. (2) in the main text, the time duration of land use right transfer (t) is 40 years in the cities of China. We obtained the spatial patterns of green spaces using exponential functions that similar to Atack and Margo (1998) and Wang (2009).

The cultural services (yuan m^{-2} urban area yr^{-1}) in a location were calculated using Eq. (3) in the main text. We obtained the spatial patterns of cultural services using logarithmic functions in regression after comparing several functions types.

Calculation for regulating services

The regulating services of urban green spaces are calculated based on data from the field survey. The biophysical values of regulating services in the three case cities are shown in Supplementary Table S1.

Carbon sequestration Urban green spaces include trees, shrubs, and herbs, while only the trees were considered to provide carbon sequestration, for the shrubs and herbs cannot sequestrate the fixed carbon for more than 50 years (IPCC, 2007). Carbon sequestration per urban land area, φC_l (ton CO_2 m^{-2} urban area yr^{-1}) was,

$$\varphi C_l = C \times CG_l \tag{1}$$

where C (ton CO_2 m^{-2} green space yr^{-1}) is $NPP \times 0.5$, NPP (ton biomass m^{-2} green space yr^{-1}) is the net primary productivity of green space; 0.5 is the carbon conversion coefficient. The NPP was the annual increment of DBH measured by tree ring samples from field surveys in the three case cities (method followed by Nowak et al., 2008). CG_l is the green space coverage in location l (see Eq. (1) in the main text) and divided by 100 for unit conversion.

Oxygen release The O_2 released by green spaces per urban land area in location l,

φO_l (ton m^{-2} yr^{-1}) was,

$$\varphi O_l = \varphi C_l \times 32/44 \quad (2)$$

where φC_l was calculated in Eq. (1).

Air filtering We calculated the amount of air filtered by urban green spaces per land area in location *l*, φAF_l (ton m^{-2} urban area yr^{-1}), based on the method of Jim and Chen (2008) and divided by urban area,

$$\varphi AF_l = AF_l \times CG_l \quad (3)$$

where AF_l is air filtering of green space (ton m^{-2} green space yr^{-1}).

Runoff mitigation We quantified rainwater runoff mitigation (φRM_l, ton m^{-2} urban area yr^{-1}) through precipitation, and the runoff coefficient to calculate the capacity (Pataki et al., 2011; Barral and Oscar, 2012) by dividing by urban area,

$$\varphi RM_l = pr \times (RI_{im} - RI_g) \times \rho \times CG_l \quad (4)$$

where RM_l is the runoff mitigation of green space (ton m^{-2} green space yr^{-1}); *pr* is the average annual precipitation; RI_{im} is the runoff rate on an impervious surface (0.83 based on Pataki et al. (2011)). RI_g is the runoff rate in an urban green space (0.13 based on Bonan (2002)); ρ is water density.

Noise reduction We calculated the noise reduction by urban green space per land area in location *l*, φNR_l (ton m^{-2} urban area yr^{-1}), based on the structure characteristics of the green spaces (Fang and Ling, 2003), and divided by urban area,

$$\varphi NR_l = NR_l \times CG_l \quad (5)$$

where NR_l is the noise reduction ability (ton m^{-2} green space yr^{-1}).

Microclimate regulation We calculated the microclimate regulation by urban green space per land area in location *l*, φMR_l (ton m^{-2} urban area yr^{-1}), which can be measured by the energy consumption savings from air conditioning (Wang et al., 2005; Yang et al., 2005), divided by urban area,

$$B_f = B_t \times \alpha \quad (6)$$

$$\varphi MR_l = (B_f \times l_p + pr \times sw) \times h \times e \times CG_l \quad (7)$$

where B_f is the leaf biomass (ton m^{-2}); B_t is the biomass of urban trees (ton m^{-2}); α is the proportion of leaf biomass in tree biomass (8.73%, Yao et al., 2003); l_p is evapotranspiration intensity (451.9 ton water per ton fresh leaf per year, here we assume that the energy will be reduced during the summer time, which is set at 3 months); *sw* is the

soil evaporation coefficient (this article takes 0.05); h is the heat consumed by vaporization of a ton of water (2.26×10^6 kJ), e is the efficiency of energy reduction from evapotranspiration (we set 10%).

Biogenic volatile organic compound (BVOC) emissions The BVOC emissions from green spaces per urban land area, $\varphi BVOC_l$ (ton C m^{-2} urban area yr^{-1}) followed the method by Chang et al. (2012) and divided by urban area,

$$\varphi BVOC_l = BVOC_l \times CG_l \tag{8}$$

where $BVOC_l$ was the emission intensity (ton m^{-2} green space yr^{-1}).

Monetizing the regulating services The values were calculated by using the biophysical values multiplied by the prices, and then calculated the sum of them,

$$RES_l = \sum_{i=1}^{i=n} \varphi BR_{il} \times PR_i \tag{9}$$

where RES_l is the regulating service (yuan m^{-2} urban area yr^{-1}) in location l; φBR_{il} is the biophysical value of regulating service i in location l (ton m^{-2} urban area yr^{-1}); n is the number of considered categories of regulating services; PR_i is the price of regulating service i (yuan ton^{-1}). The price of each regulating service was listed in Supplementary Table S6, and the monetary value was listed in Supplementary Table S7.

Data collection and re-calculation for the cultural services by CVM and HPM

Contingent valuation method (CVM) We collected the data of the total cultural services of green spaces in a city assessed in the literature (Supplementary Table S5). Since the CVM has only a total value of cultural services of the green spaces in a city, we then divided the total value by the total urban area to get the average value of cultural services per land area.

Hedonic pricing method (HPM) We collected the data of price elasticity of green spaces for house price assessed by the HPM (Supplementary Table S5). The value of cultural services of a green space in a location was calculated by multiplying the house price with the price elasticity. The datasets were not equal in size for we could only get the data of those houses that were near green spaces from existing studies.

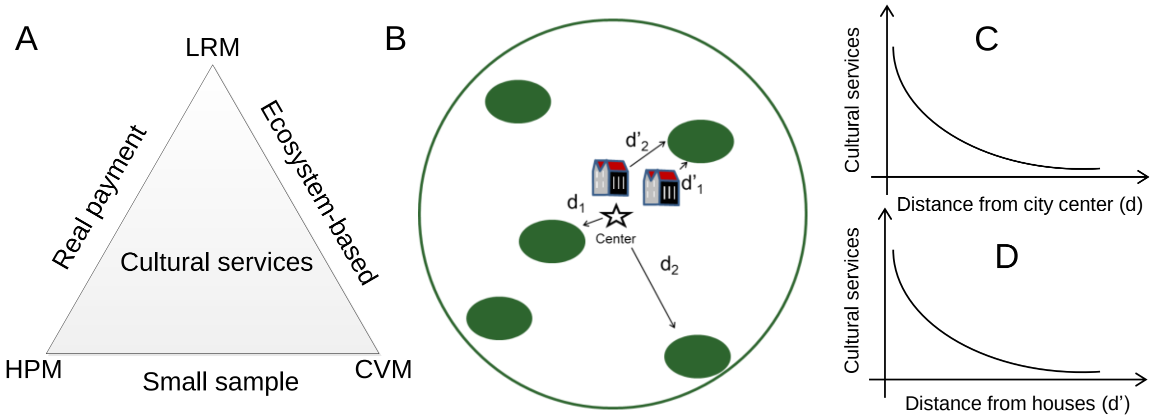

Supplementary Figure S1. Comparisons of LRM and other two existing methods on assessing the cultural services provided by green spaces. (A) the attributes of three methods, where each edge of the triangle is the similarity among the three methods: both LRM and HPM are on the real payment, both LRM and contingent valuation method (CVM) are ecosystem-based, both HPM and CVM are small samples for complex investigations; (B) in a urban area, the same area of the green spaces (green filled circles) near city center (d_1) has higher cultural services than that near urban edge (d_2) for different land rents assessed by LRM, or the cultural services of the green spaces shared by houses with different distance (d'_1 or d'_2) assessed by HPM, or the cultural services of the green spaces cannot be assessed by HPM if no commercial housing surrounded; (C) the cultural services assessed by LRM along the cenetr-edge gradient; (D) the cultural services assessed by HPM along the distance from the green spaces.

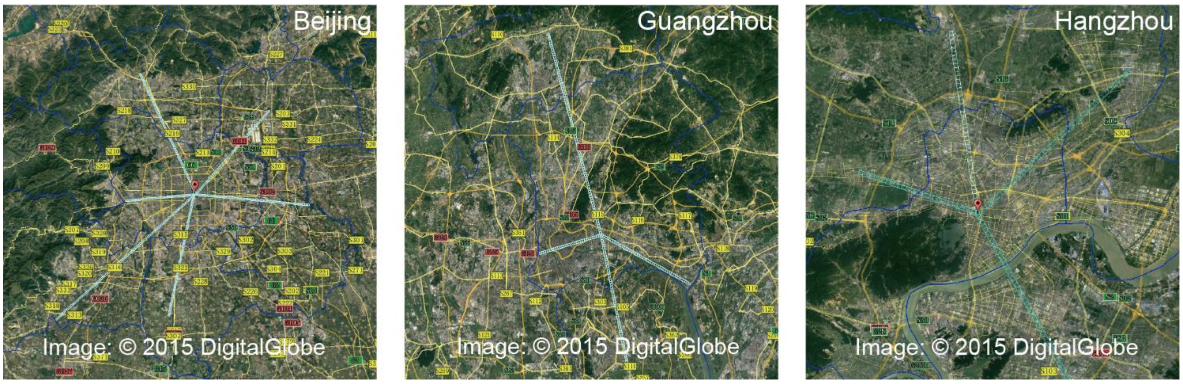

Supplementary Figure S2. Sample transects for visual interpretation. The map of China was generated in ArcGIS v10.2. The images of Beijing, Guangzhou, and Hangzhou are from Google Earth. Four sample transects (containing grid boxes of 450 m × 450 m) overlaid the built-up area in 2005 for Guangzhou and Hangzhou, and six sample transects in Beijing. All the sample transects were generated in ArcGIS v10.2.

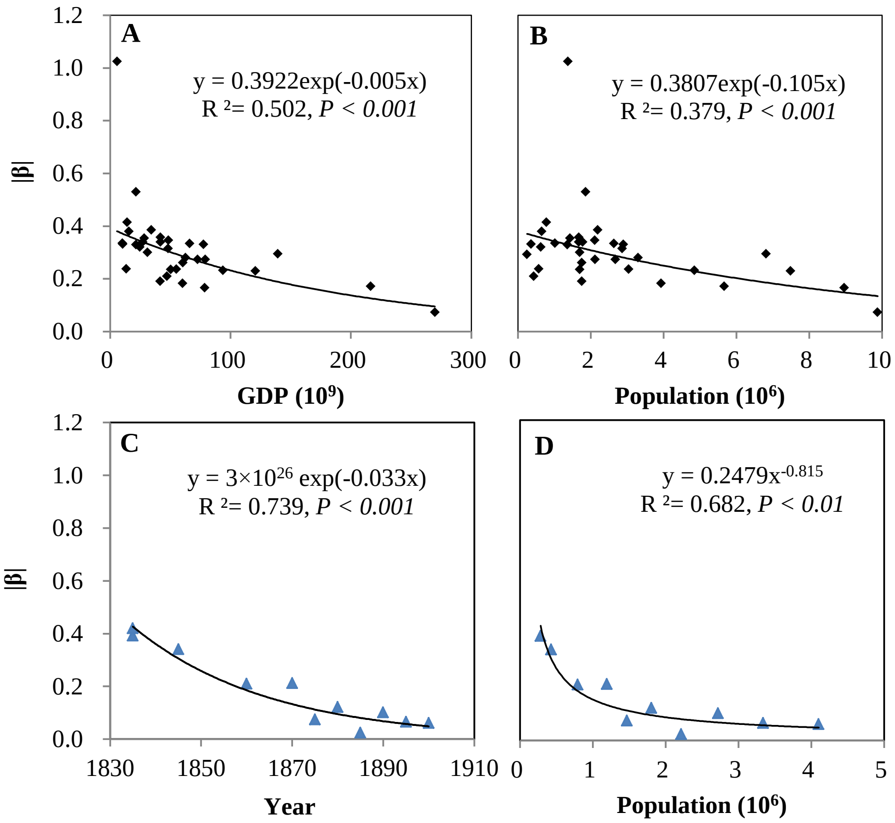

Supplementary Figure S3. Scaling of land rent patterns along the center-edge gradient in urban areas. (A) β in response to gross domestic production (GDP) of 35 cities in China, (B) population of 35 cities in China (Supplementary Table S4); (C) exponent β in response to year (1835-1910) in New York, (D) population in New York (Supplementary Table S3).

Supplementary Table S1 Biophysical value of regulating services of green spaces in the three case cities

Regulating services	Unit	Beijing	Guangzhou	Hangzhou
Carbon sequestration	ton CO_2 ha^{-1} yr^{-1}	9.86[a]	11.04[a]	15.77[a]
Oxygen release	ton O_2 ha^{-1} yr^{-1}	7.17[a]	8.03[a]	11.47[a]
Air filtering				
NO_2	ton NO_2 ha^{-1} yr^{-1}	0.026[b]	0.012[c]	0.061[a]
SO_2	ton SO_2 ha^{-1} yr^{-1}	0.020[b]	0.026[c]	0.094[a]
PM_{10}/ TSP	ton PM_{10} ha^{-1} yr^{-1}	0.150[b]	0.150[c*]	0.203[a]
Runoff mitigation	m^3 m^{-2} yr^{-1}	0.388[a]	1.183[a]	0.945[a]
Noise reduction	dBA m^{-2} yr^{-1}	0.17[a]	0.15[a]	0.18[a]
Microclimate regulation	KWh m^{-2} yr^{-1}	4.13[a]	7.03[a]	7.10[a]
BVOC emissions	ton C km^{-2}	4.49[d]	6.11[a]	3.13[e]

Note: a. Data are from our field survey; b. Yang et al., 2005; c. Jim and Chen, 2008; d. Wang et al., 2003; e. Chang et al., 2012. * Air filtering is calculated in total suspended particulate (TSP) instead of PM_{10}.

Supplementary Table S2 Commercial benchmark land price in Beijing, Guangzhou, and Hangzhou city

City	Land price section (grade)	Land price (yuan m^{-2})	Range of section (km from center)
Beijing	I	7210-9750	0-6
	II	5680-7680	0-8
	III	4530-6130	1-10
	IV	3720-5092	3-13
	V	2720-4000	6-16
	VI	1970-2900	9-26
	VII	1150-1980	16-33
	VIII	530-1180	29-36
Guangzhou	I	12715	0-5

	II	6090	2-10
	III	5100	3-11
	IV	4640	5-21
	V	3240	13-18
	VI	2760	11-23
	VII	2080	22-40
Hangzhou	I	9100	0-1
	II	6910	0-3
	III	5270	1-6
	IV	4430	2-7
	V	2850	4-8
	VI	1600	6-17
	VII	1050	9-23
	VIII	660	13-25

Note: A section has the same land price on a two-dimension pattern. However, there are overlaps between sections when measuring on a one-dimension pattern. Some land price sections out of the built-up area were not considered. The land price data were from from the Land Resources Bureau of the city or China's urban land price monitoring network (http://www.landvalue.com.cn/).

Supplementary Table S3 Land rent pattern along the center-edge gradient of the urban area in New York, USA from 1835-1900

Year	Function (y = a × exp(β*x*))	R^2
1835	y = 17.73exp(-0.392x)	0.837
1845	y = 7.62exp(-0.341x)	0.781
1860	y = 31.30exp(-0.210x)	0.989
1870	y = 95.75exp(-0.212x)	0.971
1875	y = 136.45exp(-0.075x)	0.994
1880	y = 143.98exp(-0.122x)	0.998
1885	y = 95.01exp(-0.024x)	0.990
1890	y = 156.16exp(-0.102x)	0.999
1895	y = 166.85exp(-0.066x)	0.998
1990	y = 74.18exp(-0.061x)	0.986

Data source: Atack and Margo (1998). In the function, *x* means distance from the city center (km), y means land price ($ per m^2).

Supplementary Table S4 Land rent pattern along the center-edge gradient of urban areas in 35 cities in China

City	Year	Function (y = a × exp (β*x*))	R^2
Beijing	2001	y = 15430exp(-0.073x)	0.962
Tianjin	2000	y = 10001exp(-0.295x)	0.993
Wuhan	2000	y = 5935exp(-0.230x)	0.962
Guangzhou	2000	y = 16514exp(-0.172x)	0.990
Shenyang	2000	y = 7034exp(-0.232x)	0.969
Chongqing	2000	y = 9683exp(-0.166x)	0.920
Ha'erbin	2000	y = 6445exp(-0.236x)	0.971
Nanjing	2000	y = 14830exp(-0.331x)	0.997
Xi'an	2000	y = 3550exp(-0.183x)	0.920
Chengdu	1999	y = 10504exp(-0.280x)	0.996
Changchun	1999	y = 3540exp(-0.315x)	0.919
Dalian	2000	y = 7382exp(-0.273x)	0.986
Hangzhou	1999	y = 8376exp(-0.261x)	0.979
Xuzhou	2003	y = 3961exp(-0.339x)	0.963
Nanchang	2000	y = 3958exp(-0.300x)	0.878
Suzhou	2002	y = 9260exp(-0.273x)	0.980
Yantai	2003	y = 4992exp(-0.235x)	0.989
Jinan	2000	y = 5128exp(-0.334x)	0.976
Zhengzhou	2000	y = 5316exp(-0.386x)	0.996
Kunming	2000	y = 6861exp(-0.346x)	0.999
Lanzhou	2000	y = 3961exp(-0.339x)	0.963
Changsha	2000	y = 4113exp(-0.190x)	0.916
Nanning	2000	y = 5514exp(-0.329x)	0.969
Zhuhai	2003	y = 3905exp(-0.210x)	0.979
Zhenjiang	2002	y = 4180exp(-0.320x)	0.903
Wuhu	2002	y = 3476exp(-0.380x)	0.962
Haikou	2000	y = 1749exp(-0.238x)	0.947
Neijiang	2002	y = 4180exp(-0.320x)	0.903
Ezhou	2001	y = 1656exp(-0.335x)	0.969
Jiaxing	2001	y = 4176exp(-0.415x)	0.997
Taizhou	2001	y = 4723exp(-0.354x)	0.999
Jinhua	2001	y = 2427exp(-0.332x)	0.969
Hulun Buir	2002	y = 768exp(-0.293x)	0.981

Data source: Wang (2009). In the function, *x* means distance from the city center (km), y means land price (yuan m^{-2}).

Supplementary Table S5 Data source of CVM and HPM for calculating cultural services of green spaces in the three case cities

Method	Beijing	Guangzhou	Hangzhou
Contingent valuation method (CVM)	Li et al., 2013	Jim and Chen, 2006a	Chen et al., 2006
Hedonic pricing method (HPM)	Xia et al., 2012	Jim and Chen, 2006b	Wen et al., 2012

Supplementary Table S6 Price of regulating services of green spaces in the three case cities

Regulating services	Unit	Beijing	Guangzhou	Hangzhou
Carbon sequestration[a]	yuan kg^{-1}	1.20	1.20	1.20
Oxygen release[a]	yuan kg^{-1}	1.00	1.00	1.00
Air filtering[a]	yuan kg^{-1}			
NO_2		0.63	0.63	0.63
SO_2		1.20	1.20	1.20
PM_{10}		0.15	0.15	0.15
Runoff mitigation[b]	yuan m^{-3}	3.50	1.50	1.40
Noise reduction[c]	yuan $dB(A)^{-1}$	7.66	7.66	7.66
Microclimate regulation[b]	yuan kW h^{-1}	0.45	0.61	0.53
BVOCs emission[a]	yuan kg^{-1}	5.05	5.05	5.05

Note: Data are from: a. State Forestry Administration of China (2008); b. Editorial Department of Price Yearbook of China (2006); c. Chen et al., (2011).

Supplementary Table S7 Monetary value of regulating services of green spaces in the three case cities

Regulating services	Unit	Beijing	Guangzhou	Hangzhou
Carbon sequestration	yuan m^{-2}	1.18	1.32	1.89
Oxygen release	yuan m^{-2}	0.72	0.80	1.15
Air filtering	yuan m^{-2}	0.006	0.006	0.020
Runoff mitigation	yuan m^{-2}	1.36	1.50	1.40
Noise reduction	yuan $dB(A)^{-1}$	1.30	1.15	1.37
Microclimate regulation	yuan m^{-2}	1.84	4.29	3.77
BVOCs emission	yuan m^{-2}	-0.02	-0.03	-0.02

References

Atack, J. & Margo, R. A. “Location, location, location!” The price gradient for vacant urban land: New York, 1835 to 1900. *J. Real Estate Finance.* **16**, 151-172 (1998).

Bonan, G. *Ecological climatology: concepts and applications*. (Cambridge University Press, Cambridge, NY, USA, 2002).

Chang, J. *et al*. An inventory of biogenic volatile organic compounds for a subtropical urban–rural complex. *Atmos. Environ*. **56**, 115-123 (2012).

Chen, B., Bao, Z. & Zhu, Z. Assessing the willingness of the public to pay to conserve urban green space: the Hangzhou City, China, case. *J. Environ. Health*. **69**, 26 (2006).

Chen, L., Xie, G. D. & Gai, L. Q. Research on noise reduction service of road green spaces-a case study of Beijing. *J. Nat. Resour*. **26**, 1526-1534 (2011). (in Chinese)

Editorial Department of Price Yearbook of China. Price Yearbook of China. (Huazheng Press, Beijing, China, 2006).

Fang, C. F. & Ling, D. L. Investigation of the noise reduction provided by tree belts. *Landscape Urban Plan.* **63**, 187-195 (2003).

IPCC, A. *Intergovernmental panel on climate change*. Geneva: IPCC Secretariat (2007).

Jim, C. Y. & Chen, W. Y. Recreation–amenity use and contingent valuation of urban greenspaces in Guangzhou, China. *Landscape Urban Plan.* **75**, 81-96 (2006a).

Jim, C. Y. & Chen, W. Y. Impacts of urban environmental elements on residential housing prices in Guangzhou (China). *Landscape Urban Plan.* **78**, 422-434 (2006b).

Jim, C. Y. & Chen, W. Y. Assessing the ecosystem service of air pollutant removal by urban trees in Guangzhou (China). *J. Environ. Manage.* **88**, 665-676 (2008).

Li, Q. Y., Shi, M. M. & Wang, M. Z. Estimate the value of green space through CVM method. *Sci. Technol. Guide.* **20**, 66-67 (2013). (in Chinese)

State Forestry Administration of China. Specifications for Assessment of Forest Ecosystem Services in China. (Standards Press, Beijing, China, 2008).

Wang, X. M., Li, Z., Jiang, X. & Liao, W. B. Quantitative evaluation on the ecological benefit of public park green space. *J. Plant Resour. Environ.* **14**, 42-45 (2005). (in Chinese with English abstract)

Wang, R. The structure of Chinese urban land prices: Estimates from benchmark land price data. *J. Real Estate Finance.* **39**, 24-38 (2009).

Wang, Z. H., B, Y. H. & Zhang, S. Y. A biogenic volatile organic compounds emission inventory for Beijing. *Atmos. Environ.* **37**, 3771-3782 (2003).

Wen, H. Z., Li, X. N. & Zhang, L. Impacts of the urban landscape on the housing price: A case study in Hangzhou. *Geogr. Research.* **31**, 1806-1814 (2012). (in Chinese)

Xia, B., Zhang, B. & Xie, G. D. The value-added effect of park green space on residential property in Beijing. *Resour. Sci.* **34**, 1347-1353 (2012). (in Chinese)

Yang, J., McBride, J., Zhou, J. & Sun, Z. The urban forest in Beijing and its role in air pollution reduction. *Urban For. Urban Green.* **3**, 65-78 (2005).

ESRI. ArcGIS version 10.2 (GIS by ESRI, 2013). Available at: https://www.arcgis.com (Accessed 22th October 2015).
